# Development and Evaluation of a Point-of-Care Test in a Low-Resource Setting with High Rates of Chlamydia trachomatis Urogenital Infections in Fiji

**DOI:** 10.1128/JCM.00182-21

**Published:** 2021-06-18

**Authors:** Deborah Dean, Sumeetha Swaminathan, Mike Kama, Sophie Goemans, Daniel Faktaufon, Noor Alnabelseya, Dawn Spelke, Kamin Kahrizi, Matthew Black, Debkishore Mitra

**Affiliations:** a Department of Medicine, University of California, San Francisco, California, USA; b Department of Pediatrics, University of California, San Francisco, California, USA; c Department of Bioengineering, University of California, San Francisco, California, USA; d Department of Bioengineering, University of California, Berkeley, California, USA; e Bixby Center for Global Reproductive Health, University of California, San Francisco, California, USA; f Fiji Centre for Communicable Disease Control, Ministry of Health and Medical Services, Suva, Fiji; g Lucira Health, Emeryville, California, USA; Marquette University

**Keywords:** *Chlamydia trachomatis*, limit of detection, low-resource setting, point-of-care test, sexually transmitted infections

## Abstract

Rapid and precise detection of Chlamydia trachomatis, the leading global cause of sexually transmitted infections (STI), at the point of care (POC) is required for treatment decisions to prevent transmission and sequelae, including pelvic inflammatory disease, ectopic pregnancy, tubal factor infertility, and preterm birth. We developed a rapid POC test (POCT), termed LH-POCT, which uses loop-mediated amplification (LAMP) of nucleic acids. We performed a head-to-head comparison with the Cepheid Xpert CT/NG assay using clinician-collected, deidentified paired vaginal samples from a parent study that consecutively enrolled symptomatic and asymptomatic females over 18 years of age from the Ministry of Health and Medical Services Health Centers in Fiji. Samples were processed by the Xpert CT/NG assay and LH-POCT, blinded to the comparator. Discrepant samples were resolved by quantitative PCR. Deidentified clinical data and tests for Trichomonas vaginalis, *Candida*, and bacterial vaginosis (BV) were provided. There were a total of 353 samples from 327 females. C. trachomatis positivity was 16.7% (59/353), while the prevalence was 16.82% (55/327) after discrepant resolution. Seven discrepant samples resolved to four false negatives, two false positives, and one true positive for the LH-POCT. The sensitivity of the LH-POCT was 93.65% (95% confidence interval [CI], 84.53% to 98.24%), and specificity was 99.31% (95% CI, 97.53% to 99.92%). Discrepant samples clustered among women with vaginal discharge and/or BV. The prototype LH-POCT workflow has excellent performance, meeting many World Health Organization ASSURED criteria for POC tests, including a sample-to-result time of 35 min. Our LH-POCT holds promise for improving clinical practice to prevent and control C. trachomatis STIs in diverse health care settings globally.

## INTRODUCTION

Chlamydia trachomatis is a human pathogen and the leading cause of bacterial sexually transmitted infections (STIs) worldwide (1; https://www.cdc.gov/nchhstp/newsroom/2019/2018-STD-surveillance-report.html). Untreated C. trachomatis can lead to significant morbidity and mortality due to pelvic inflammatory disease (PID), infertility, ectopic pregnancy, preterm birth, and infant pneumonitis, in addition to increasing the risk of HIV transmission (2–7). These diseases reach beyond the individual to adversely impact national and global economies as well as sociobehavioral dynamics within communities (8–10).

The annual rates of C. trachomatis STIs have been increasing steadily for the last decade according to the World Health Organization (WHO) and the CDC (11; https://www.cdc.gov/nchhstp/newsroom/2019/2018-STD-surveillance-report.html). Global estimates of C. trachomatis STIs are approximately 130 million cases per year [11; https://www.who.int/en/news-room/fact-sheets/detail/sexually-transmitted-infections-(stis)], with the highest concentrations among the Pacific Island Countries and Territories (PICT) of the Western Pacific Ocean, where over 61 million individuals are infected (12, 13). However, this number underestimates the true incidence and prevalence, because screening for C. trachomatis is not routinely performed in low- to middle-income countries (LMIC) that comprise most of the countries in the PICT. This is due primarily to the cost and supply chain issues related to commercial diagnostics, not to mention the lack of infrastructure, including reliable electricity, in many of these countries. Commercially available nucleic acid amplification tests (NAAT) for C. trachomatis require expensive equipment, collection and detection kits, and a trained technician.

The WHO recently endorsed a global health care strategy to eliminate the threat of STIs by the year 2030 (12). To achieve this goal, diagnostic tests need to not only be available and cost appropriate but also should provide results within a reasonable time frame to inform treatment. Most patients are unwilling to wait longer than 40 min, although longer wait times in LMICs appear to be acceptable (14, 15). An unwillingness to wait increases the risk that patients will be lost to follow-up. Current NAATs take hours or a day or more for results (16, 17). While some C. trachomatis diagnostic tests take only 15 to 30 min, they are based on immunoassays that have an unacceptably low sensitivity of 17% to 83% (17, 18) and are not recommended for use by the WHO (17).

The WHO ASSURED (Affordable, Sensitive, Specific, User-friendly, Rapid, Equipment free, and Delivered to users) criteria were created as a guide for developing point-of-care tests (POCT) (19). In addition, nucleic acid-based tests are considered essential for POCTs according to the CDC and the WHO (20, 21). Near-patient tests for C. trachomatis have been developed or are in the pipeline but still require equipment and some technical training, with results from ∼60 min to 6 h (17, 22, 23). POCTs in the pipeline are promising (17, 24). However, many rely on instrumentation with four or more steps. Further, there are no or limited data on comparisons with commercial NAATs to determine their sensitivity and specificity.

We report a prototype nucleic acid-based POCT workflow for C. trachomatis that uses isothermal loop-mediated amplification (LAMP) of nucleic acids in conjunction with a colorimetric readout chemistry, monitored in real time by a portable, low-cost instrument with on-board optical detection elements, obviating the need for expensive thermocycling and fluorescent instrumentation. The sample-to-result time is less than 35 min for our C. trachomatis LH-POCT, including about 5 min of hands-on time. Here, our C. trachomatis LH-POCT was compared to the commercial Cepheid Xpert CT/NG assay using deidentified clinical vaginal samples from participants in LMIC Fiji to assess the sensitivity, specificity, and accuracy of our assay.

## MATERIALS AND METHODS

### Study design, population, and sample collection.

The study was cross-sectional in design, with nonprobability consecutive sampling of females over 18 years of age seen in Fijian Ministry of Health and Medical Services (MoHMS) health centers and outreach locations in the Central Division of Viti Levu, Fiji, as described in the parent study (25). IRB approval was obtained from UCSF and the Fijian MoHMS in accordance with the Declaration of Helsinki for the parent study. Participants consented and were enrolled, completed a sociodemographic questionnaire, provided information on symptoms, and underwent a pelvic exam along with vaginal swab collection, as described previously (25). The current study used deidentified participant information with a unique ID number (i.e., no trace to patient name or personal identifiers) for all data, including demographics, signs, symptoms, and coinfection.

FLOQswabs (Copan, Murietta, CA) were used to simultaneously collect two mid-vaginal samples by trained clinicians as described previously (25). Briefly, one swab was placed in transport medium (SWAB/A-50 collection kit; Cepheid, Sunnyvale, CA) for subsequent Xpert CT/NG testing by the GeneXpert system (Cepheid), and the other swab was placed back into the original dry tube swab container without medium for the C. trachomatis LH-POCT. Both were transported the same day to the Fiji Communicable Disease Clinical Laboratory for processing. A third swab was collected for vaginal pH and wet preparations to determine the presence of Trichomonas vaginalis, *Candida*, and clue cells as described previously (25). Three or more Amsel criteria were used to diagnose bacterial vaginosis (BV): vaginal pH of ≥4.5, homogeneous vaginal discharge, fishy amine odor when KOH was applied to vaginal material on a glass slide, and >20% clue cells on wet preparation (26).

### Identification of primer sets for LAMP of C. trachomatis.

A semiautomated bioinformatic pipeline for screening 230 available complete C. trachomatis genomes, representing a diversity of strains, was used to identify conserved targets for primer set design for LAMP assays. Briefly, the commercial CLC Genome Workbench and open-source tools inGAP (27) and SSAHA-SNP (28) were used to output lists of predicted variants to Excel. Single-nucleotide polymorphisms (SNP) and indels in each genome common to at least two methods were retained and used in downstream analysis. From these data, the data set was mined *in silico* to identify high-copy-number targets for conserved genomic regions to detect all reference and clinical C. trachomatis strains. Genomes from all other STI and genital commensal species based on the FDA list for cross-reactivity testing were also pulled from public databases and aligned to the genomes.

Candidate target sequences were selected that had sufficient divergence from cross-reactive species and satisfied other requirements, such as GC content and number of mutations to design primer sets. We focused on rRNA targets due to their higher gene copy number (2 copies/genome) and high levels of expression. Several primer sets were designed, and each was validated *in silico* for C. trachomatis specificity by BLAST. Primer sets were also designed to detect a synthetic plant-based DNA segment (not present in clinical samples) that was used as an internal amplification control (IC) that amplifies if no inhibitors are present.

### Screening against spiked C. trachomatis controls, sexually transmitted pathogens, and commensals.

The designed primer sets were tested against genome equivalents of ≥10^5^ bacteria/ml and ≥10^5^ 50% tissue culture infective doses/ml (viruses) for STIs and other potential urogenital pathogen species (Neisseria gonorrhoeae, Trichomonas vaginalis, Mycoplasma genitalium, human papillomavirus, herpes simplex virus, Candida albicans, Gardnerella vaginalis, Bacteroides fragilis, Mobiluncus curtisii, and Mycoplasma hominis) and 50 common urogenital species (see Table S1 in the supplemental material). All 18 reference strains of C. trachomatis (1) were also screened to ensure that all strains would be detected. C. pneumoniae and C. psittaci were screened given the potential cross-reactivity based on *in silico* analyses. The final C. trachomatis primer sets did not cross-react with any species and were used in all subsequent tests.

### Determination of LOD.

To test the limit of detection (LOD) of the assay, a stock solution of C. trachomatis reference strain Da was quantified for chlamydial genome copy numbers by our in-house quantitative PCR (qPCR) (29) assay, using a standard curve based on 10-fold serial dilutions of a linearized plasmid containing the single-copy C. trachomatis gene *ompA*. One genome constitutes one elementary body (EB), which is the infectious particle of Chlamydia. The Da strain was serially diluted to generate a target concentration of 14, 1.4, and 0.14 EBs per reaction. A proprietary lysis buffer containing the EBs was kept at room temperature for 5 min. The lysate (30 µl) was then added to lyophilized pellets containing the C. trachomatis-specific primer sets and the thermolabile amplification reagents. The final concentrations were 466.7 EBs/ml, 46.7 EBs/ml, and 4.7 EBs/ml. The reaction mix was amplified by LAMP, and a successful LOD was achieved when ≥95% (≥19/20) of replicates were detected.

### Screening of LAMP assays for inhibitory substances.

Interfering substances, expected to be present in vaginal swab samples, were tested at specific concentrations: 1%, vol/vol, for blood; 1%, wt/vol, for mucin; 0.5%, vol/vol, for semen; and 10^6^ cells/ml for leukocytes. The substances were added to the lysis buffer at the concentrations indicated. This lysis buffer was then aliquoted into the IC assay and independently into the C. trachomatis assay in triplicate and tested per the typical workflow.

### LH-POCT workflow.

The C. trachomatis LH-POCT workflow involves sample preparation, amplification with real-time detection, and, finally, analysis. Sample preparation involves eluting the swab in a proprietary lysis buffer, which contains the colorimetric detection reagent, by swirling for 10 s. The swab is then discarded and the lysis buffer tube is capped, inverted three times, and left at room temperature for 5 min. Thirty microliters of each lysate was then added to four individual PCR tubes that each contained a single lyophilized pellet. For each patient sample, four PCR tubes were used: two PCR tubes contain identical pellets for detecting C. trachomatis, and two PCR tubes contain identical pellets for the IC. Identical duplicates of each pellet were used to improve performance and reduce overall failure rates. Following rehydration of the pellets, the PCR tubes were placed in a portable instrument to measure the change in reaction color over time. The time to result (TTR) was determined when the absolute signal increased by 50% relative to the signal at 5 min. All time cutoffs were set to 25 min. A test was considered positive if either of the C. trachomatis pellets were positive, invalid if both IC pellets were negative, and negative if at least one IC pellet was positive and C. trachomatis pellets were negative.

### Discrepant resolution.

The reference standard was defined as the Xpert CT/NG assay result (30). When the Xpert and C. trachomatis LH-POCT results were in agreement, no additional testing was performed. For discrepant samples where the LH-POCT did not agree with the Xpert result, DNA was extracted from both original remnant samples (i.e., Cepheid collection medium and LH-POCT preprocessed buffer) using a QIAamp DNA minikit (Qiagen, Germantown, MD) per the manufacturer’s protocol. An in-house qPCR (29) was used for discrepant resolution where the initial results were masked. Briefly, standard curves were calculated based on 10-fold serial dilutions of a linearized plasmid containing the single-copy C. trachomatis gene *ompA* and a linearized plasmid containing the single-copy β-actin gene. The respective copy number was calculated based on the standard curve for each plasmid. The reference standard for discrepant analysis was the agreement of two out of the three test results.

### Data analysis.

The study was powered for a minimum of 50 C. trachomatis-positive samples based on an estimated overall prevalence of 15% for the age range in this study (25) and ∼330 female participants. The presumed sensitivity of the C. trachomatis LH-POCT was 94% with a 98% specificity compared to commercial NAATs, based on in-house testing of archived clinical samples for estimates of 95% confidence intervals (CI) of 84.34% to 98.82% and 96.93% to 99.78%, respectively. Only participants with data on signs, symptoms, coinfections, and Xpert and C. trachomatis LH-POCT results were included in the analyses. Calculations of sensitivity, specificity, and 95% CI were determined based on the reference standard. Pearson chi-square test was used for comparisons with age, ethnicity, symptomatic versus asymptomatic presentations, clinical signs, and coinfection. Analyses were performed in R, version 3.5.0 (31).

## RESULTS

### Participant characteristics.

The overall prevalence of C. trachomatis among the participants was 16.82% (55/327), counting only their baseline visit; 26 participants were seen and tested more than once, with 59 (16.71%) positive tests for 353 samples. Women under 25 years had the highest prevalence at 22.86%. Table 1 shows the data on participant characteristics and C. trachomatis POCT, Xpert, and discrepant results in addition to coinfection results for the baseline visit only. Of the 12 patients who were over 40 years, seven were in their 40s, four in their 50s, and one was 64 years old. The majority of the participants were of iTaukei-Fijian ethnicity.

**TABLE 1:** Participant demographics, symptoms and signs, and coinfection data for C. trachomatis LH-POCT, Xpert CT/NG, and discrepant results^*a*^

Demographics	Total (*N* = 327) (%)	C. trachomatis positive (*N* = 55) (%)	Discrepants (*N* = 7)	No. of discrepant results for:	
				C. trachomatis-positive LH-POCT	C. trachomatis-positive Xpert
Age, in yr					
18–24	70 (21.41)	16 (22.86)	1	0	1
25–30	101 (30.89)	18 (17.82)	2	1	1
31–40	144 (44.04)	21 (14.58)	4	2	2
>40	12 (3.67)	0 (0)	0	0	0
Ethnicity					
iTaukei Fijian	184 (56.27)	44 (23.91)	5	3	2
Indo-Fijian	90 (27.52)	2 (2.22)	1	0	1
Other	53 (16.21)	9 (16.98)	1	0	1
Clinic					
Women's wellness health clinic	245 (74.92)	40 (16.33)	6	3	3
Reproductive health clinic	5 (1.53)	1 (20.00)	0	0	0
University clinic 1	30 (9.17)	6 (20.00)	1	0	1
University clinic 2	16 (4.89)	5 (31.25)	0	0	0
Outreach clinics	12 (3.67)	0 (0.00)	0	0	0
Valelevu health clinic	19 (5.81)	3 (15.79)	0	0	0
Asymptomatic	123 (37.61)	22 (17.89)	0	0	0
Symptomatic	200 (61.16)	33 (16.50)	7	3	4
Missing	4 (1.22)	0 (0)	0	0	0
Symptoms					
Dysuria	36	7	0	0	0
Urgency	32	7	1	1	0
Frequency	37	11	1	1	0
Hematuria	8	1	0	0	0
Lower abdominal pain	93	16	1	1	0
Dyspareunia	45	9	1	0	1
Bleeding with intercourse	24	4	0	0	0
Cramping	51	9	1	0	1
Vaginal discharge	85	14	2	0	2
Missing	4	0	0	0	0
No signs	163 (50.46)	23 (14.11)	4	1	3
Signs	160 (49.54)	32 (20.00)	3	2	1
Missing	4 (1.22)	0 (0)	0	0	0
Signs					
Cervical discharge	95	21	0	0	0
Vaginal discharge	116	26	2	2	0
Both	60	15	1	0	1
Cervical motion tenderness	22	7	0	0	0
Missing	4	0	0	0	0
No coinfection	229 (70.03)	32 (13.97)	5	3	2
Coinfection	98 (29.97)	23 (23.47)	2	0	2
Any coinfections					
N. gonorrhoeae	15	11	0	0	0
T. vaginalis	15	3	0	0	0
* Candida*	31	2	0	0	0
BV	55	14	2	0	2
≥2 coinfections	17	6	0	0	0

a Because a participant may have more than one symptom, sign, or coinfection, the total per column may be more than 327.

The majority of discrepant findings were relatively evenly distributed between C. trachomatis LH-POCT and Xpert results for each category in Table 1. However, there were two discrepant results for the Xpert assay for women with vaginal discharge and two for the LH-POCT for women with BV.

### Inhibitory substances and determination of LOD.

Table 2 shows the data obtained from the serial dilutions of C. trachomatis organisms added to the lysis buffer. At 1.4 EBs/reaction (equivalent to 46.7 EBs/ml), C. trachomatis was reliably detected in less than 25 min. This compares favorably to the Xpert CT/NG claimed LOD of 84 to 161 EBs/ml in K121710 according to the package insert. The IC and C. trachomatis assays were positive in less than 25 min when spiked with 1% (vol/vol) blood, 1% (wt/vol) mucin, 0.5% (vol/vol) semen, and 10^6^ cells/ml for leukocytes.

**TABLE 2:** LOD for C. trachomatis LAMP primer set assays for spiked-in lysis buffer

Parameter	No. of EBs/ml	No. positive/no. tested	TTR ± SD (min) of positive reactions
No. of EBs/reaction			
14	466.7	3/3	14.2 ± 1.9
1.4	46.7	3/3	16.3 ± 2.6
0.14	4.7	1/3	15.8 ± NA^*a*^
LOD confirmation			
1.4	46.7	20/20	14.2 ± 1.2

a Not applicable.

### Accuracy of the C. trachomatis POCT.

The results comparing the C. trachomatis POCT with the Xpert CT/NG assay after discrepant resolution by qPCR are shown in Table 3. Of the 353 samples, there were a total of 7 (1.98%) discrepant samples that were further analyzed by qPCR for C. trachomatis using both Xpert remnant and LH-POCT preprocessed collection media; the qPCR results were similar for both media. One discrepant was positive by the C. trachomatis LH-POCT and qPCR but not the Xpert assay; this sample was considered a true positive. For the remaining six discrepant samples, the Xpert CT/NG assay matched the qPCR results: four Xpert assay-positive results and two Xpert assay-negative results (Table 3).

**TABLE 3:** Comparative performance of C. trachomatis LH-POCT with Xpert CT/NG test after C. trachomatis qPCR discrepant resolution on clinical vaginal samples^*a*^

C. trachomatis LH-POCT result	C. trachomatis Xpert result (no.)		
	Positive	Negative	Total
Positive	59	2	61
Negative	4	288	292
Total	63	290	353

a Accuracy, 98.30% (95% CI, 96.34% to 99.37%); sensitivity, 93.65% (95% CI, 84.53% to 98.24%); specificity, 99.31% (95% CI, 97.53% to 99.92%).

Eleven samples had invalid IC results and, therefore, were not included in the above-described analyses (i.e., 364 samples minus 11, for 353 total analyzed samples). These 11 samples were negative by both the LH-POCT and Xpert CT/NG assay. Amplification was documented for the Xpert internal positive control for these 11 samples, although the range was 28 to 35 cycles, suggesting that some inhibition was present in samples showing a higher number of cycles at the start of amplification.

## DISCUSSION

The global burden of C. trachomatis STIs is extremely high and climbing. LMICs in the Western Pacific have been shown to be at especially high risk for STIs. However, the PICT lacks affordable NAATs that could substantially improve detection and treatment to decrease transmission and reduce upper genital tract sequelae. We have developed a rapid and inexpensive C. trachomatis LH-POCT workflow that could be deployed to LMICs in the PICT but also to rural, inner-city, STI, family planning, and adolescent clinics worldwide for testing and appropriate treatment at the POC.

POCTs that do not require a laboratory setting are needed, but many have not been tested in the patient setting. In this study, we compared our LH-POCT workflow against the Xpert CT/NG assay in the clinical laboratory, and both tests were run the same day of collection to avoid any variation that might occur from storage at 4°C or a longer time interval before running the tests. Our LH-POCT workflow showed a sensitivity and specificity of 93.65% and 99.31%, respectively, compared to the Xpert CT/NG assay after discrepant resolution, with an overall accuracy of 98.30%. This sensitivity is within the range of current commercial NAATs that are at ∼90% to 100% (32). A recent study modeling C. trachomatis transmission among sex partners based on U.S. data indicated that a POCT sensitivity of as low as 90% coupled with increased screening could substantially reduce C. trachomatis incidence and prevalence in addition to lowering cases of PID (33). In support of this finding, a recent editorial stressed the need for “access to POC tests (for C. trachomatis) as soon as possible, even if their performances are slightly below those of lab-based assays” (32). Furthermore, while it would be optimal to have a combined N. gonorrhoeae and C. trachomatis POCT, and there have been recent advances in POC STI testing with the *binx io* system (binx health, Inc.) (34) that provides results in 30 min, we have focused on developing a rapid C. trachomatis POCT that could be deployed immediately to meet the global public health crisis related to C. trachomatis STIs. Additionally, there is a need for more POC testing solutions that provide results in less than 40 min, and we are currently developing a dual N. gonorrhoeae and C. trachomatis POCT to meet that need.

While we did not utilize our C. trachomatis LH-POCT workflow in the actual clinics, the ease of use would likely limit disruption in patient flow: three steps, portable instrumentation, and results in 35 min (i.e., 5 min of hands-on time, 5 min for sample lysis, and 25 min for amplification). Nonetheless, it will be important for our and other POCTs to be field tested in the actual clinical settings where they are intended for use. Moreover, a sample-to-result time of less than 40 min would further reduce infection rates, since a number of studies have shown that patients are willing to wait for that length of time and be treated if they are positive (14, 15, 35).

Our comparator was one reference NAAT and an in-house qPCR assay for discrepant testing. This design is similar to other recent studies that have used only one reference test (24, 36, 37). While ideally the study design would have included two reference tests (38) and our current design may have introduced some bias, there was high concordance between the Xpert CT/NG assay and our C. trachomatis LH-POCT results. Furthermore, discrepant analysis included DNA extraction from both remnant samples (i.e., Cepheid collection media and LH-POCT preprocessed buffer) with similar qPCR results.

We only used vaginal swabs in this study, as they are highly acceptable for both self- and physician collection (21). Furthermore, the vaginal site reflects endocervical infection, which poses the greatest risk for upper genital tract disease (36; https://www.cdc.gov/std/chlamydia/stdfact-chlamydia-detailed.htm). While urine samples are also easy to obtain, they do not always correlate with cervical infection. Indeed, in a number of studies, cervical C. trachomatis infection has been detected in 10% to 30% of women who had no urethral or bladder infection (21, 39, 40). However, urine samples would also be an optimal sample type for testing males, and we are currently expanding our test to include this specimen type.

Our LH-POCT was able to detect C. trachomatis across all age groups and from both symptomatic and asymptomatic women with and without signs of an STI, and those with coinfections. While most discrepant samples were evenly distributed across the categories in Table 1, the highest number (i.e, two discrepant results) was found for the sign and symptom of vaginal discharge and the presence of BV. For symptoms, our C. trachomatis LH-POCT missed two true-positive samples for vaginal discharge, while the Xpert assay missed two true positives for the sign of vaginal discharge. It is reasonable to assume that this type of material contains inhibitors. Similarly, with BV, where two true positives were missed by our C. trachomatis LH-POCT, some inhibitors may be present. These findings provide additional useful information for refining our LH-POCT to eliminate any potential inhibitors from clinical samples.

While we excluded from our analyses all samples with an invalid LH-POCT IC, the four false-negative samples may also be due, in part, to a low level of inhibitory factors, which did not impact IC performance. If inhibitors are the culprit, we should see improvements using various mitigation strategies that include changes to the elution volume and variation in enzyme concentrations used for amplification. These changes may also reduce the overall occurrence of invalid IC results. These factors will be taken into consideration as our LH-POCT workflow is migrated onto our instrument-free platform.

Our prototype C. trachomatis LH-POCT is promising for use in developed countries and LMICs, as it fulfills many of the WHO ASSURED test criteria: sensitive, specific, user-friendly, rapid, and equipment free. In addition, knowledge that a C. trachomatis POCT is available will likely lead to increased screening and utilization of the test in traditional as well as nontraditional settings, such as family planning clinics, prisons, emergency rooms, free clinics that serve the homeless, and, eventually, pharmacies. In addition, the assay technology is compatible with an instrument-free platform that we have developed that lends itself to at-home testing, a paradigm shift in molecular testing. The availability of at-home solutions has shown clear benefits to health care and cost reductions across many disease areas (41, 42). In the infectious disease space, rapid over-the-counter (OTC) test kits for HIV have encouraged testing among those who otherwise would not have been tested (43). At-home detection of C. trachomatis can similarly benefit from this approach and encourage testing among individuals who would avoid clinic visits due to logistical burdens or privacy concerns.

Currently, only about 50% of the target population is being screened in the United States (https://www.cdc.gov/std/chlamydia/hedis.htm). Broad screening will aid in reducing the stigma or hesitancy in screening, which will help to normalize health care-seeking behavior related to STIs (32). With further development and testing at the POC along with evaluation of the workflow at the patient/provider level, our C. trachomatis LH-POCT holds promise to significantly impact the control of C. trachomatis STIs by increasing screening and treatment and decreasing patient loss to follow-up, thereby interrupting the transmission of C. trachomatis STIs and the global prevalence of their devastating sequelae.
