# Supplementary material for: Development and Evaluation of a Point-of-Care Test in a Low-Resource Setting with High Rates of Chlamydia trachomatis Urogenital Infections in Fiji
Source: J Clin Microbiol. 2021 Jun 18;59(7):e00182-21. doi: 10.1128/JCM.00182-21 (PMC8218753; doi:10.1128/JCM.00182-21)
Supplement: Supplemental file 1 — . Download JCM.00182-21-s0001.pdf, PDF file, 65 KB [file jcm.00182-21-s0001.pdf]

Supplemental Information

Development and evaluation of a Point-of-Care Test (POCT) in a low resource setting with high rates of *Chlamydia trachomatis* urogenital infections in Fiji

Deborah Dean[a,b,c,d,e#], Sumeetha Swaminathan[b], Mike Kama[f], Sophie Goemans[b,g], Daniel Faktaufon[f], Noor Alnabelseya[g], Dawn Spelke[g], Kamin Kahrizi[g], Matthew Black[g], Debkishore Mitra[g#]

[a]Department of Medicine, University of California, San Francisco, CA, USA

[b]Department of Pediatrics, University of California, San Francisco, CA, USA

[c]Department of Bioengineering, University of California, San Francisco, CA, USA

[d]Department of Bioengineering, University of California, Berkeley, CA, USA

[e]Bixby Center for Global Reproductive Health, University of California, San Francisco, CA, USA

[f]Fiji Centre for Communicable Disease Control, Ministry of Health and Medical Services, Suva, Fiji

[g]Lucira Health, Emeryville, CA, USA

TABLE S1. Sexually transmitted, commensal and other pathogenic species screened for cross-reactivity with *C. trachomatis* primer sets.

**Sexually transmitted organisms**	**Organisms involved in urogenital pathogenesis**	**Commensal Species**	**Other**
Neisseria gonorrhoeae	*Candida albicans*	*Entercoccus faecalis*	*Chlamydia pneumoniae*
Human papilloma virus	*Bacteriodes ureolyticus*	*Escherichia coli*	*Chlamydia psittaci*
Herpes simplex virus 1	*Bacteriodes fragilis*	*Haemophilus influenzae*	
Herpes simplex virus 2	*Gardnerella vaginalis*	*Klebsiella pneumoniae*	
Trichomonas vaginalis	*Mycoplasma hominis*	*Lactobacillus acidophilus*	
	Ureaplasma urealyticum	*Listeria monocytogenes*	
	Mobiluncus curtisii	*Micrococcus luteus*	
	Bacillus subtilis	*Peptostreptococcus anaerobius*	
	Mycoplasma genitalium	*Pseudomonas aeruginosa*	
		Staphylococcus aureus	
		Streptococcus pneumoniae	
		Vibrio parahaemolyticus	
		Yersinia enterocolitica	
		Alcaligenes faecalis	
		Arcanobactrium poygenes	
		Candida tropicalis	
		Coryebacterium genitalium	
		Human Cytomegalovirus	
		Lactobacillus delbrukkii	
		Legionella pneumophila	
		Mycobacterium smegmatis	
		Neisseria mucosa	
		Providencia stuartii	
		Pseudomonas putida	
		Rhodospirillum rubrum	
		Saccharomyces cerevisiae	
